# Differential thermal stability, conformational stability and unfolding behavior of Eis proteins from *Mycobacterium smegmatis* and *Mycobacterium tuberculosis*

**DOI:** 10.1371/journal.pone.0213933

**Published:** 2019-03-25

**Authors:** Shashi Anand, Arsheed Ahmad Ganaie, Charu Sharma

**Affiliations:** CSIR-Institute of Microbial Technology, Chandigarh, India; Russian Academy of Medical Sciences, RUSSIAN FEDERATION

## Abstract

Eis (Enhanced Intracellular Survival) is an important aminoglycoside N-acetyltransferase enzyme contributing to kanamycin resistance in Mtb clinical isolates. Eis proteins from *M*. *tuberculosis* (RvEis) and *M*. *smegmatis* (MsEis) have 58% identical and 69% similar amino acid sequences and acetylate aminoglycosides at multiple amines. Both the Eis proteins are hexameric and composed of two symmetric trimers. RvEis has remarkable structural stability and heat-stable aminoglycoside acetyltransferase activity. Although the structure and biochemical properties of MsEis have been studied earlier, the detailed characterization of its acetyltransferase activity and structural stability is lacking. In this study, we have performed comparative analysis of structural stability and aminoglycoside acetyltransferase activity of RvEis and MsEis proteins. Unlike RvEis, MsEis undergoes a three-state unfolding induced by heat or chemical denaturants and involves self-association of partially unfolded oligomers to form high molecular weight soluble aggregates. MsEis is highly susceptible to chemical denaturants and unfolds completely at lower concentrations of GdmCl and urea when compared to RvEis. In contrast to RvEis, the oligomeric forms of MsEis are SDS sensitive. However, SDS treatment resulted in increased helix formation in MsEis than RvEis. MsEis shows lesser thermostable activity with a decreased efficiency of kanamycin acetylation in comparison to RvEis. Furthermore, overexpression of MsEis does not provide thermal resistance to *M*. *smegmatis* unlike RvEis. Collectively, this study reveals that homologous proteins from pathogenic and nonpathogenic mycobacteria follow different modes of unfolding and demonstrate differential structural stability and activity despite highly similar sequences and oligomeric organization.

## Introduction

The acetylation of aminoglycosides by aminoglycoside acetyltransferases is one of the important mechanisms contributing to drug resistance in tuberculosis [1]. These aminoglycoside acetyltransferases belong to pervasive GCN5-related*N*-acetyltransferase (GNAT) super family [2]. A number of aminoglycoside acetyltransferase enzymes involved in drug resistance in different pathogenic bacteria have been characterized [3–6].

Eis (Rv2416c, Enhanced Intracellular Survival protein) protein from *M*. *tuberculosis* (RvEis) is one such GNAT enzyme that acetylates multiple amines of aminoglycoside drugs and renders them nonfunctional [7,8]. However, Eis proteins with ~ 75% identity to RvEis are present in members of *M*.*tuberculosis* complex (MTC) like *M*. *africanum*, *M*. *bovis*, *M*. *canettii*, *M*. *marinum*. Besides, *M*. *smegmatis*, a non pathogenic mycobacterial species, also expresses Eis homolog with 58% amino acid sequence identity to RvEis [9]. Eis homologues have also been identified in non-mycobacterial pathogens like *Bacillus anthracis* and *Anabaena variabilis* which can acetylate many aminoglycosides [10,11].

Several studies have been performed for the structural and biochemical characteristics of Eis proteins from *M*. *smegmatis* (MsEis) and *M*. *tuberculosis* (RvEis) [8,9,12]. RvEis and MsEis possess similar hexameric structure [12]. The narrow and elongated channel in the active site of RvEis enables effective utilization of both aminoglycosides as well as lysine residues in polypeptide substrates for acetylation. On the contrary, a deep and round shaped substrate binding pocket of MsEis allows acetylation of bulkier aminoglycosides and N-terminal amino group of peptide substrates [12]. MsEis has similar acetylation pattern as that of RvEis for most of the aminoglyosides except apramycin, paromomycin and hygromycin [9]. There are contrasting reports on differences in the aminoglycoside acetyltransferase activities of RvEis and MsEis proteins. According to Garneau-Tsodikova’s group, the substrate binding affinity and catalytic efficiency of RvEis for kanamycin, paromomycin and amikacin acetylation is higher than that of MsEis [6,10,11]. On the other hand, Suh’s group suggested that MsEis has an increased binding affinity towards kanamycin, amikacin and paromomycin and a higher catalytic efficiency than RvEis [12]. Thus, the comparison of aminoglycoside acetyltransferase activities of MsEis and RvEis proteins remains controversial.

RvEis has a high structural stability and thermostable acetyltransferase activity [13]. A glycine-rich loop present near the active site of RvEis is important for its thermal and structural stability [14]. However, biophysical features of MsEis protein remain uncharacterized. Therefore, to understand the molecular and structural basis of differences in the acetylation efficiencies of RvEis and MsEis, we performed in-depth comparative analysis of thermostability, conformational stability, and acetyltransferase activity of RvEis and MsEis proteins. We observed that MsEis protein differs in structure stability from RvEis and forms molten-globule like intermediates of high molecular weight during unfolding induced by heat or chemical denaturants. However, the thermal denaturation of both MsEis and RvEis proteins is irreversible. While MsEis undergoes partial unfolding accompanied by the formation of soluble aggregates, RvEis protein unfolds completely followed by exothermal precipitation. Besides, MsEis exhibits lesser binding affinity and reduced catalytic efficiency towards kanamycin in comparison to RvEis.

## Materials and methods

### Materials

Enzymes used in the cloning or modification of the DNA were procured from Thermo Fisher scientific. All the routine reagents and chemicals were purchased from Sigma-Aldrich unless specified. Chromatography columns and markers for gel filtration chromatography were purchased from GE Healthcare Life Sciences, USA.

### Cloning, expression and purification of MsEis protein

The gene encoding *M*. *smegmatis* Eis (MSMEG_3513) protein was amplified by PCR from *M*. *smegmatis* genomic DNA using forward primer 5’-TGCGAATTCATGATCACGCCGCGCACCCTTCAC-3’ and reverse primer 5’-CCGCTCGAGTCAGAATCCGTATCCCAGCTCT-3’ in which the underlined nucleotides represent EcoRI and XhoI restriction sites, respectively. The amplified PCR product was cloned into pET-28a vector at EcoRI and XhoI sites so as to express *M*. *smegmatis* Eis (MsEis) protein with hexahistidine tag at N-terminus. The overexpression and purification of MsEis was carried out as described for RvEis [15]. Similarly, *M*. *tuberculosis* Eis (RvEis) protein was purified using the previously cloned construct pET-28a-RvEis[15]. Elution fractions containing desired protein as analyzed on SDS-PAGE gel were pooled and dialyzed against 20 mM sodium phosphate buffer (pH 8.0) and concentrated using 10K MWCO Pierce^TM^ concentrators. The yield of purified MsEis protein was 4 mg per liter of culture.

### Circular dichroism spectrometry

Circular dichroism (CD) measurements were performed on Jasco J-815 spectropolarimeter calibrated with ammonium (+)-10-Camphorsulfonate. The far- UVCD spectra of 3 μM MsEis protein equilibrated with 20 mM phosphate buffer (pH 8.0) was recorded in 1 mm cell at 25°C. The results are expressed as MRE (Mean Residue Ellipticity) in deg.cm^2^.dmol^-1^, calculated using the following equation:

$$\left[\text{MRE}\right]=\;\theta \times 100\times {\text{M}}_{\text{r}}/\;\text{c}\times \text{d}\times {\text{N}}_{\text{A}}$$

where θ is the observed ellipticity in degrees, c = protein concentration in mg/ml, and d = path length in cm, M_r_ = Protein molecular weight and N_A_ = number of amino acids. For analysis of thermal stability, 5 μM of protein sample was incubated from 25°C to 100°C (as determined by sample internal probe) and changes in the secondary structure were monitored by measuring changes in ellipticity at 222 nm using 3°C/min heating rate. For the scan-rate dependence experiment, 3 μM of either MsEis or RvEis protein was used to monitor heat induced changes in CD ellipticity at 222 nm from 25°C to 100°C using 0.5°C/min, 1°C/min and 1.5°C/min scanning rate. For guanidium hydrochloride (GdmCl), urea and SDS induced unfolding studies, protein samples were incubated with respective denaturant for 12 h before recording the spectra from 250 nm to 200 nm wavelength. Fraction of folded protein corresponding to observed mean residual ellipticity at 222 nm was calculated using the following equation:

$$\left({\left[\theta \right]}^{\text{obs}}-{\left[\theta \right]}^{\text{den}}\right)/\left({\left[\theta \right]}^{\text{nat}}-\;{\left[\theta \right]}^{\text{den}}\right)$$

where [θ]^obs^ is the experimental observed mean residue ellipticity at 222 nm, [θ]^nat^ and [θ]^den^ are mean residue ellipticities at 222 nm when the protein is in native and fully denatured state, respectively. The obtained data points were fitted to three-state model (biDose-response curve of nonlinear regression analysis) using OriginPro 8 software (Origin Lab, Northampton, USA).

### Fluorescence spectroscopy

Intrinsic tryptophan fluorescence spectra were recorded on Varian spectrofluorimeter using 1.0 cm path length quartz cell. The emission spectrum was recorded from 300 nm to 450 nm with 5 nm slit width following excitation at 280 nm wavelength. For unfolding and refolding experiments, 1 μM of MsEis protein was treated with varying concentrations of GdmCl and urea for 2 h, 4 h, 6 h, 8 h, 10 h and 12 h time intervals. At 0 h, intrinsic tryptophan fluorescence spectra was recorded immediately after adding the respective denaturant. For the refolding analysis at different time intervals, MsEis treated with either GdmCl or urea was extensively dialyzed in 20 mM Phosphate buffer (pH 8.0) before recording emission spectra. For comparative analysis of stability of MsEis and RvEis proteins, 1μM of either MsEis or RvEis protein was treated with GdmCl or urea for 12 h before recording the emission spectra. The baseline recorded using buffer alone was subtracted from fluorescence values recorded for sample.

The effect of temperature on the emission spectra of either RvEis or MsEis was analyzed by incubating respective protein samples at different temperatures ranging from 25°C to 100°C for 10 min. For measuring effect of SDS, respective protein samples were treated with increasing concentration of SDS at 25°C for 4 h before recording fluorescence. The values corresponding to native Eis protein without any treatment were considered as 100%. OriginPro 8.0 was used for fitting the data to either two-state or three-state denaturation curve (Dose-response or biDose-response curve) using non-linear regression analysis.

### Size exclusion chromatographic analysis

Size exclusion chromatography offers the best way to study molten-globule like intermediates formed during the unfolding of proteins [16]. Therefore, in order to determine the oligomeric status of intermediates appeared during unfolding of RvEis or MsEis protein, size exclusion chromatography was performed using 100 μg of either MsEis or RvEis protein treated with 0.75 M GdmCl or 1.5 M urea for 12 h at 4°C or heated at 65°C for 10 min. After respective treatments, protein samples were centrifuged at 12,000 rpm for 10 min before loading onto Superose 6 Increase 10/300 GL column pre-equilibrated with buffer containing 10 mM Tris-Cl (pH 8.0), 150 mM NaCl, 1 mM EDTA, 0.5 mM β-ME and either 0.75 M GdmCl or 1.5 M urea and the column was run at 25°C with a flow rate of 0.5 ml/min. The column was calibrated using standard molecular weight markers under the native conditions (without denaturant) or in the presence of 0.75 M GdmCl or 1.5 M urea. Thyroglobulin (669 kDa), ferritin (440 kDa), β-amylase (200 kDa), alcohol dehydrogenase (ADH) (150 kDa), ovalbumin (43 kDa) and cytochrome c (12.9 kDa) were used as molecular weight markers. Void volume was determined using Blue dextran (2000 kDa).

### ANS binding fluorescence

For ANS (8-Anilino-1-naphthalenesulfonic acid) binding assay, 10 μM MsEis protein sample treated with increasing concentration of GdmCl and urea for 12 h at 4°C was then incubated with ANS in 20 mM phosphate buffer (pH 8.0) in a molar ratio of 1:5 for 5 min at 25°C.The spectra were recorded in dark. The ANS fluorescence emission spectra was measured from 400 nm to 600 nm following excitation at 360 nm. The slit widths were set at 5 nm for both the excitation and emission. For studying the effect of temperature on binding of ANS to MsEis protein, samples were incubated at increasing temperatures ranging from 25°C to 100°C for 10 min before the measurements were made.

### SDS-PAGE assay

10 μg of either boiled or unboiled MsEis and RvEis proteins were electrophoresed on 8% SDS-PAGE gel with sample loading dye containing 1% SDS, 0.125 M Tris (pH 6.8), 40% glycerol as described earlier [17]. Protein bands were visualized after staining with Coomassie Brilliant Blue R250 stain.

### Differential scanning calorimetry

Differential scanning calorimetry experiment was carried out using Microcal VP-DSC/ Malvern instruments (Northampton, MA, USA). Both MsEis and RvEis proteins were extensively dialyzed against 20 mM sodium phosphate buffer (pH 8.0) and concentrated so as to achieve a final concentration ranging between 0.25–1 mg/ml. The desired protein sample and dialysis buffer were filtered using 0.2 μm PVDF membrane filter (Uniflo^TM^, GE healthcare Life science, UK) and thoroughly degassed at 20°C prior to loading in calorimetric sample and reference cells having a volume capacity of ~ 0.5 ml. Thermal scans were performed using 60°C/h scan rate with 5 min prescan thermostat. For the scan-rate dependence experiment, 3 μM of either MsEis or RvEis protein was used to monitor changes in the heat capacity (Cp) at 0.5°C/min, 1°C/min and 1.5°C/min scanning rate. Between the two protein scans, the sample cell was extensively washed and tested for water-water scans. Origin 7 built-in software installed in the computer attached with the instrument was used to analyze the excess heat capacity profiles of the protein samples.

### Aminoglycoside acetyltransferase activity assays

Aminoglycoside acetyltransferase activity of RvEis and MsEis was analyzed by spectrophotometer based assay using kanamycin (KAN) and Ellman’s reagent (DTNB) as described previously [6,18]. The enzymatic reaction (100 μl) containing 2 mM DTNB, 500 μM Acetyl CoA, and 0.5 μM of either RvEis or MsEis protein in 20 mM phosphate buffer (pH 8.0) was initiated by the addition of 100 μM KAN. The acetyltransferase activity of Eis protein was determined by measuring the formation of 2-nitro-5-thiobenzoate (NTB^-^), a yellow colored product that absorbs at 412 nm (ε_412 nm_ = 14,150 M^-1^cm^-1^). The rate of product formation was assessed by following the changes in the absorbance at 412 nm per unit time over a linear range. The effect of temperature on enzymatic activity of Eis protein was evaluated by incubating respective protein samples at temperatures ranging from 20°C to 80°C for 10 min followed by centrifugation at 12,000 g for 5 min and recording the absorbance. The enzymatic activity of MsEis and RvEis at different temperatures was determined over a linear range. All the enzymatic reactions at each temperature and time interval were set up in triplicates.

### Steady-state kinetic parameters

The kinetic parameters of MsEis and RvEis proteins were determined using different concentrations of Kanamycin (20, 50, 250, 500 and 1000 μM) in the acetyltransferase reaction. The reaction was performed in a reaction mixture of 100 μl containing 2 mM DTNB, 0.5 μM of either MsEis or RvEis protein in 20 mM phosphate buffer (pH 8.0) at 25°C for 20 min. The enzymatic reaction was initiated after the addition of kanamycin and all the reactions were prepared atleast in duplicates. The reaction was monitored as described above. The kinetic parameters of both the proteins were determined by non-linear regression data fitting to Michaelis-Menten equation (Hyperbl function of Hyperbola category) using Origin® Pro 8 (Origin Lab, Northampton, USA).

### Effect of temperature on the *in vitro* growth of *M*. *smegmatis* overexpressing RvEis or MsEis

The *eis* gene from *M*. *smegmatis* and *M*. *tuberculosis* was cloned in pMV261 vector mycobacterial shuttle vector and *M*. *smegmatis* competent cells were transformed with these DNA constructs. *M*. *smegmatis* harboring vector alone was used as a negative control. Transformed bacterial cultures were grown in 7H9 media containing kanamycin (50 μg/ml) at 37°C until the OD_600_ reached 1.0. The respective bacterial cells were heated for 10 min at 37°C, 50°C, 60°C and 70°C in water bath after transferring the cultures in pre-heated glass tubes. The viability of bacterial cells after exposure to heat was determined by plating appropriate dilutions of all the treated cultures and controls in triplicates on 7H11 agar plates containing kanamycin (50 μg/ml). The colony forming units (CFUs) were counted after incubation at 37°C for 3 days.

## Results

### Secondary structural changes in MsEis protein upon temperature, GdmCl and urea induced unfolding

**Fig 1:**
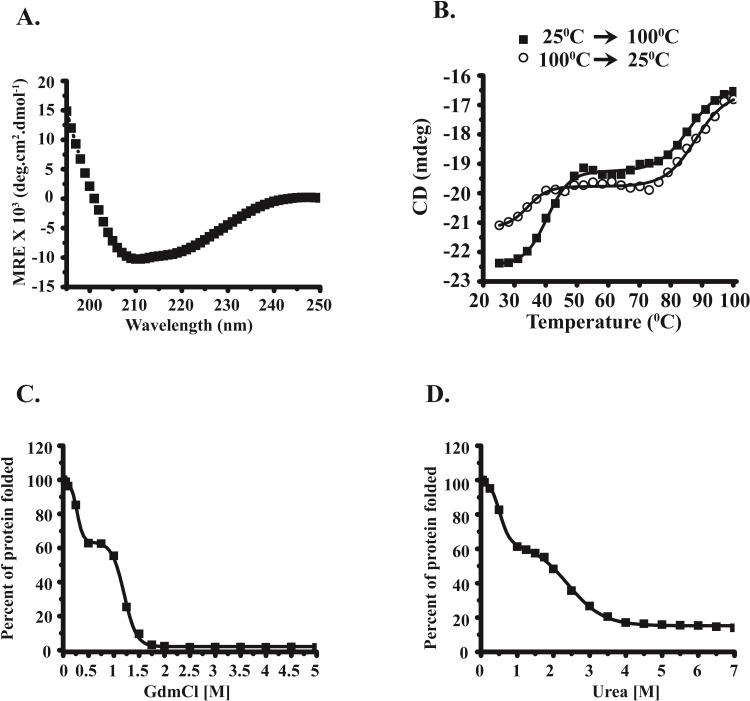
Secondary structural changes of MsEis protein in the presence of temperature, GdmCl and urea. **(Panel A)** A characteristic Far-UV CD spectra of MsEis was recorded in 20 mM phosphate buffer, pH 8.0, ranging from 250 nm to 195 nm at 25°C. (**Panel B)** Thermal melting curve of MsEis protein following changes in the ellipticity at 222 nm wavelength. (**Panel C)** Changes in the secondary structure of MsEis protein were monitored by following the changes in ellipticity at 222 nm at increasing concentration of GdmCl. (**Panel D)** Unfolding of MsEis was also monitored by following the changes in ellipticity at 222 nm wavelength at increasing concentration of urea. The value corresponding to native MsEis without any treatment was taken as 100%.

The N-terminal His tagged MsEis protein was purified using Ni-NTA affinity chromatography (S1 Fig). In far-UV CD spectra of proteins, α-helix shows two minima at 222 nm and 208 nm and β-sheets depicts a single minimum at 216 nm [19]. Far-UV CD spectra of MsEis protein indicates the presence of α-helix and β-sheets in the secondary structure as observed at 25°C (Fig 1A). Analysis of CD spectra using BestSel software [20] revealed that MsEis has 69.7% α-helix, 16% β-sheets and 14.3% other secondary structural elements including turns or random coils. Thermal melting curve analysis of MsEis protein characterized by changes in ellipticity at 222 nm with increasing temperature displayed a biphasic denaturation curve. MsEis protein began to lose its structure following incubation at 35°C, an intermediate state was formed between 50°C and 75°C and no change in the ellipticity was observed in this temperature range (Fig 1B). However, further change in ellipticity was observed when MsEis protein was incubated beyond 75°C up to 100°C. Moreover, upon cooling of MsEis protein, the second transition observed between 75°C and 100°C was reversible while the first transition observed between 25°C and 45°C was largely irreversible (Fig 1B). GdmCl and urea induced changes in the secondary structure of MsEis protein were analyzed by monitoring changes in the ellipticity at 222 nm at increasing concentrations of GdmCl and urea. GdmCl induced denaturation of MsEis is a three state process with an intermediate state formed between 0.5 M and 1.0 M GdmCl. However, two sharp sigmoidal transitions in ellipticity were observed; first between 0 M and 0.5 M and second between 1.0 M and 2.0 M GdmCl and no signal was observed above 2.0 M GdmCl (Fig 1C). Similarly, urea induced unfolding of MsEis also follows a three state denaturation process with an intermediate state formed between 1.0 M and 1.8 M urea. Moreover, ~ 80% of protein was observed to lose its secondary structure at 4 M urea (Fig 1D). The C_m_ (denaturant concentration where 50% denaturation of protein is observed) values of ~ 1.2 M and ~ 1.9 M were calculated for GdmCl and Urea induced unfolding of MsEis protein, respectively.

### Unfolding and Refolding kinetics of MsEis protein

**Fig 2:**
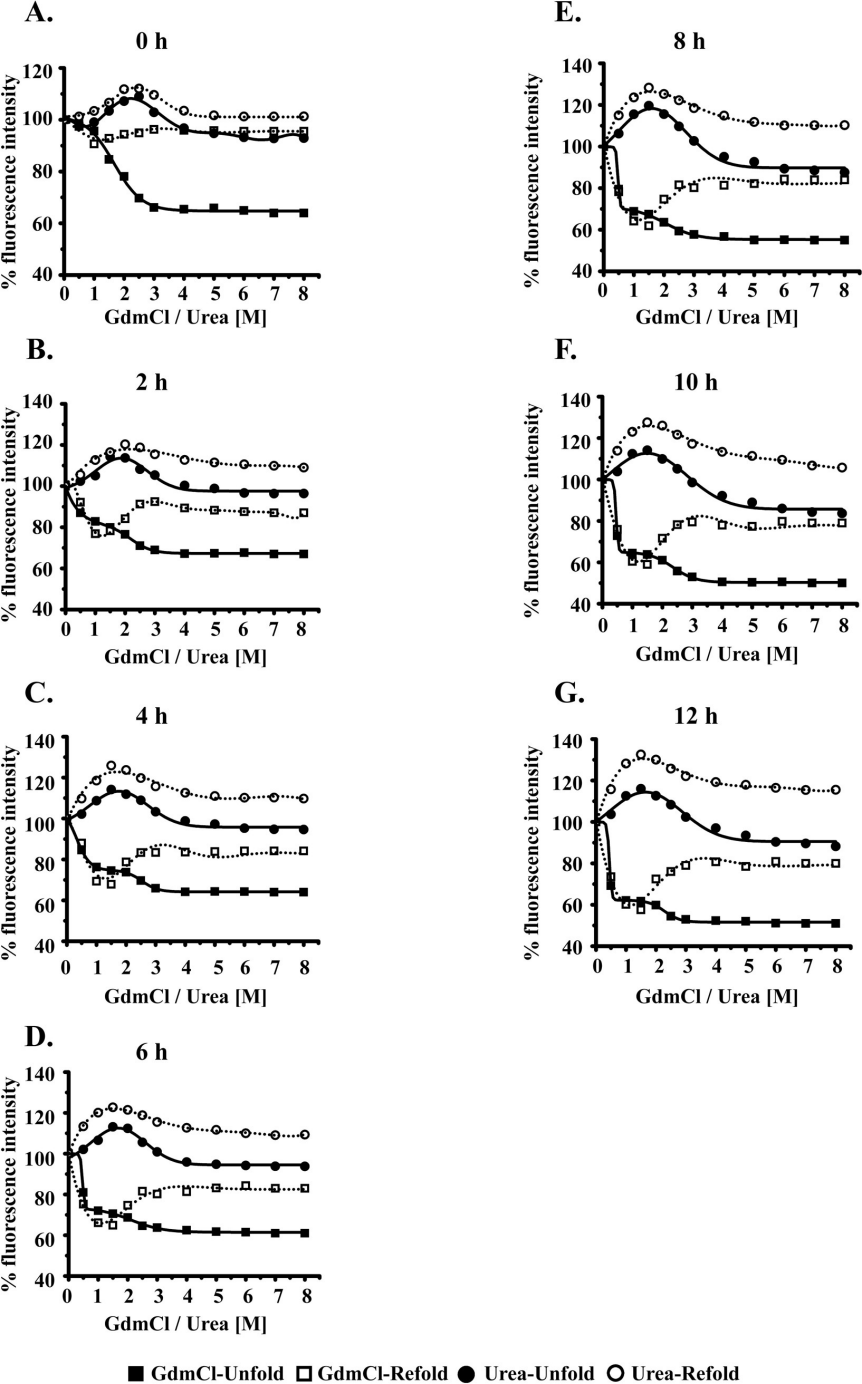
Unfolding and refolding kinetics of MsEis protein. Intrinsic tryptophan fluorescence spectra of either unfolded or refolded MsEis protein in the presence or absence of GdmCl and urea at different time intervals. 0 h **(Panel A)**, 2 h **(Panel B)**, 4 h **(panel C)**, 6 h **(Panel D)**, 8 h **(Panel E)**, 10 h **(Panel F)** and 12 h **(Panel G)**. The values corresponding to native MsEis without treatment were considered as 100% in each case of unfolding or refolding. The values obtained from emission maxima were fitted using OriginPro 8.0 software.

The comparison of amino acid sequences of MsEis and RvEis proteins is shown in S2 Fig. Both MsEis and RvEis proteins have six and seven tryptophan residues, respectively. Therefore, the tryptophan residues were used as intrinsic probe to detect the conformational changes in MsEis and RvEis proteins as a fucntion of GdmCl or urea. At 0 h, GdmCl induced unfolding of MsEis followed a two state denaturation without any intermediate formation. MsEis protein was stable upto 1 M GdmCl and undergoes a sigmoidal transition between 1M and 3 M without any further change in fluorescence intensity upto 8 M GdmCl (Fig 2A). An intermediate state that started to appear between 0.5 M and 1.5M GdmCl at 2 h was stabilized by 4.0 h and remained unchanged upto 12 h (Fig 2B–2G). The removal of respective amount of GdmCl from denautured MsEis indicated that the unfolding of MsEis was irreversible at higher concentration of GdmCl, i.e., from 2.0 M to 8.0 M. However, the intermediate state of MsEis protein that appeared between 0.5 M and 1.5 M GdmCl appeared to be reversible (Fig 2A–2G).

The urea induced denaturation of MsEis led to increased fluorescence intensity. The urea concentration at which maximum flourescence intensity was observed shifted from at 2.5 M at 0 h to 2 M and 1.5 M at 2 h and 4 h, respectively, beyond which no change was observed upto 12 h (Fig 2A–2G). Similar to unfolding, refolding of urea-treated MsEis protein also revealed enhanced fluorescence intensity indicating the presence of intermediates as observed during unfolding (Fig 2A–2G). These results indicate that the refolding of MsEis protein does not alter the intermediate state that appeared during unfolding induced by either GdmCl or urea.

### Reduced conformational stability of MsEis in comparison to RvEis

**Fig 3:**
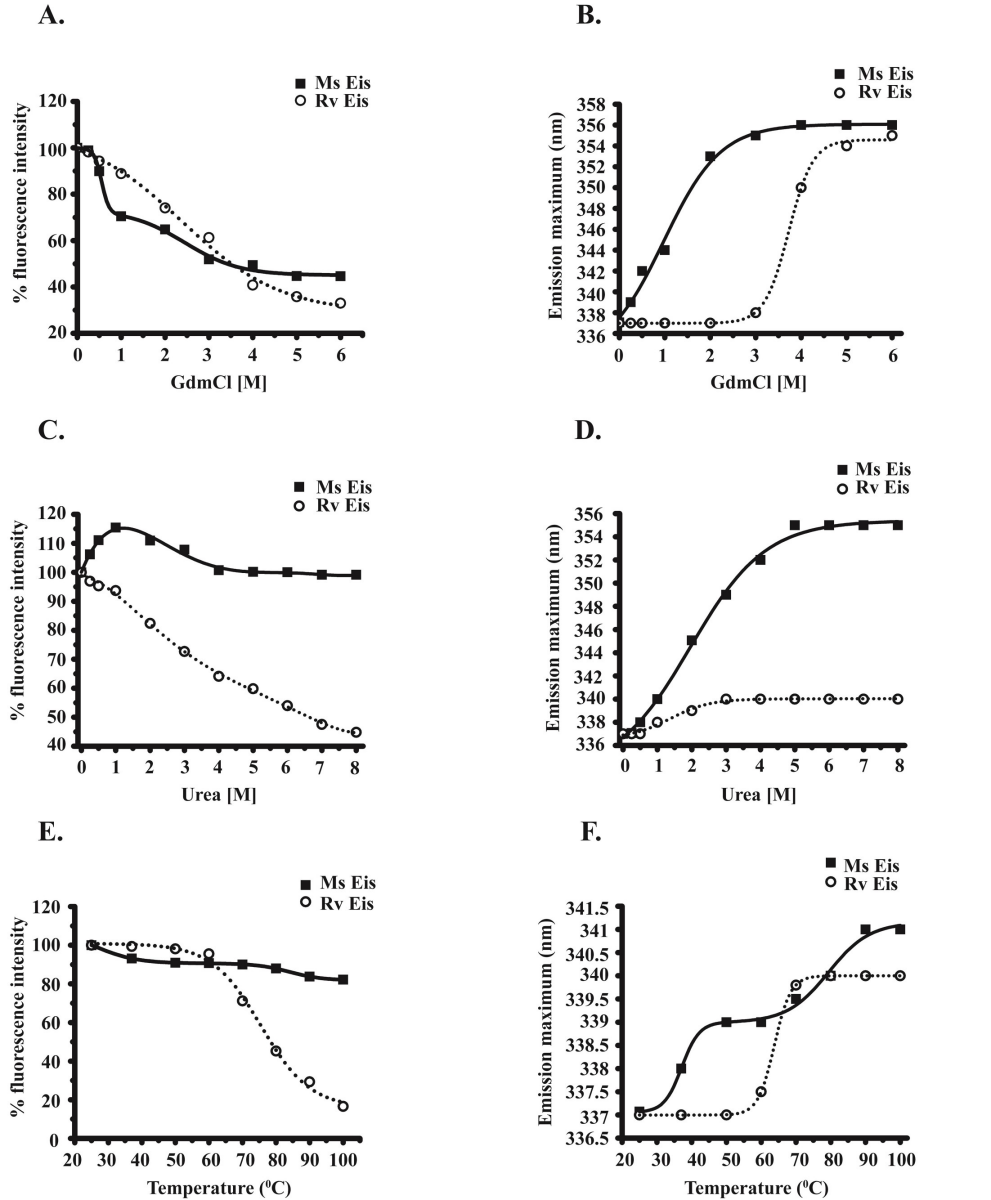
Comparison of conformational stability of MsEis and RvEis proteins in the presence of different denaturants. Intrinsic tryptophan fluorescence spectra of MsEis and RvEis proteins in the presence of increasing concentration of GdmCl **(Panel A),** urea **(Panel C)** and at increasing temperature from 25°C to 100°C **(Panel E)**. Unfolding of MsEis and RvEis proteins measured in terms of changes in emission wavelength maxima in the presence of GdmCl **(Panel B),** urea **(panel D)** and temperature **(Panel F)**. The values corresponding to native Eis without treatment were considered as 100%. Fitting of the values obtained from emission maxima were done by using equations with OriginPro 8.0 software.

Fluorescence spectroscopy was used to study the changes in the conformation and dynamic properties of MsEis and RvEis proteins in the presence of different denaturants such as GdmCl, urea and temperature. While GdmCl induced unfolding of MsEis was found to be a three-state process with two transitions, RvEis unfolding is a two-state process with single transition in fluorescence intensity observed between 1 M and 4 M GdmCl (Fig 3A). In case of MsEis, first sharp transition was observed between 0 M and 1 M GdmCl with 30% decreased fluorescence intensity and another transition between 2 M and 4 M GdmCl with only ~ 50% decrease in fluorescence intensity (Fig 3A). At 3 M GdmCl, a prominent red shift in λ_max_ from 337 nm to 355 nm was also observed for MsEis protein which was absent in case of RvEis protein (Fig 3B). In the presence of urea, the fluorescence intensity of MsEis protein was increased by 15% at 1.0 M urea followed by a decrease of 15% up to 4.0 M urea. RvEis protein follows a single sigmoidal transition in fluorescence intensity from 1.0 M to 8.0 M urea (Fig 3C). There was no change in fluorescence intensity at 8 M urea for MsEis whereas a decrease of ~ 65% intensity was observed in case of RvEis protein (Fig 3C). Further, significant increase in red shift by 18 nm (from 337 nm to 355 nm) was observed in case of MsEis whereas a red shift of only 3 nm (from 337 nm to 340 nm) was seen for RvEis (Fig 3D).

Temperature induced unfolding of MsEis protein showed very less decrease in fluorescence intensity and ~ 90% of the fluorescence intensity was retained at 100°C. Apparently, it is also a three-state transition process where an intermediate state was formed between 50°C to 75°C (Fig 3E). On the contrary, a two-state sigmoidal decrease in fluorescence intensity was observed for RvEis protein. RvEis was stable upto 60°C followed by a sharp decrease in fluorescence intensity and only ~ 20% fluorescence was retained at 100°C (Fig 3E). Furthermore, the wavelength maxima of MsEis also followed two transitions; first transition includes a shift from 337 nm to 339 nm between 30°C and 50°C and the second transition was observed from 339 nm to 341 nm between 65°C and 90°C. While in case of RvEis a single transition in λ_max_ from 337 nm to 340 nm was observed between 55°C to 70°C (Fig 3F).

### Enhanced ANS fluorescence at intermediate state during unfolding of MsEis

ANS (8-Anilino-1-naphthalenesulfonic acid) has a greater affinity for the partially unfolded or intermediate forms of proteins [21,22]. Thus, ANS was used to investigate the intermediate states that are observed during unfolding of MsEis protein. ANS in the presence of buffer shows a characteristic emission spectra (λ_max_ = 518 nm). The addition of MsEis protein resulted in an enhanced fluorescence intensity of ANS accompanied by a blue shift in emission maxima.

**Fig 4:**
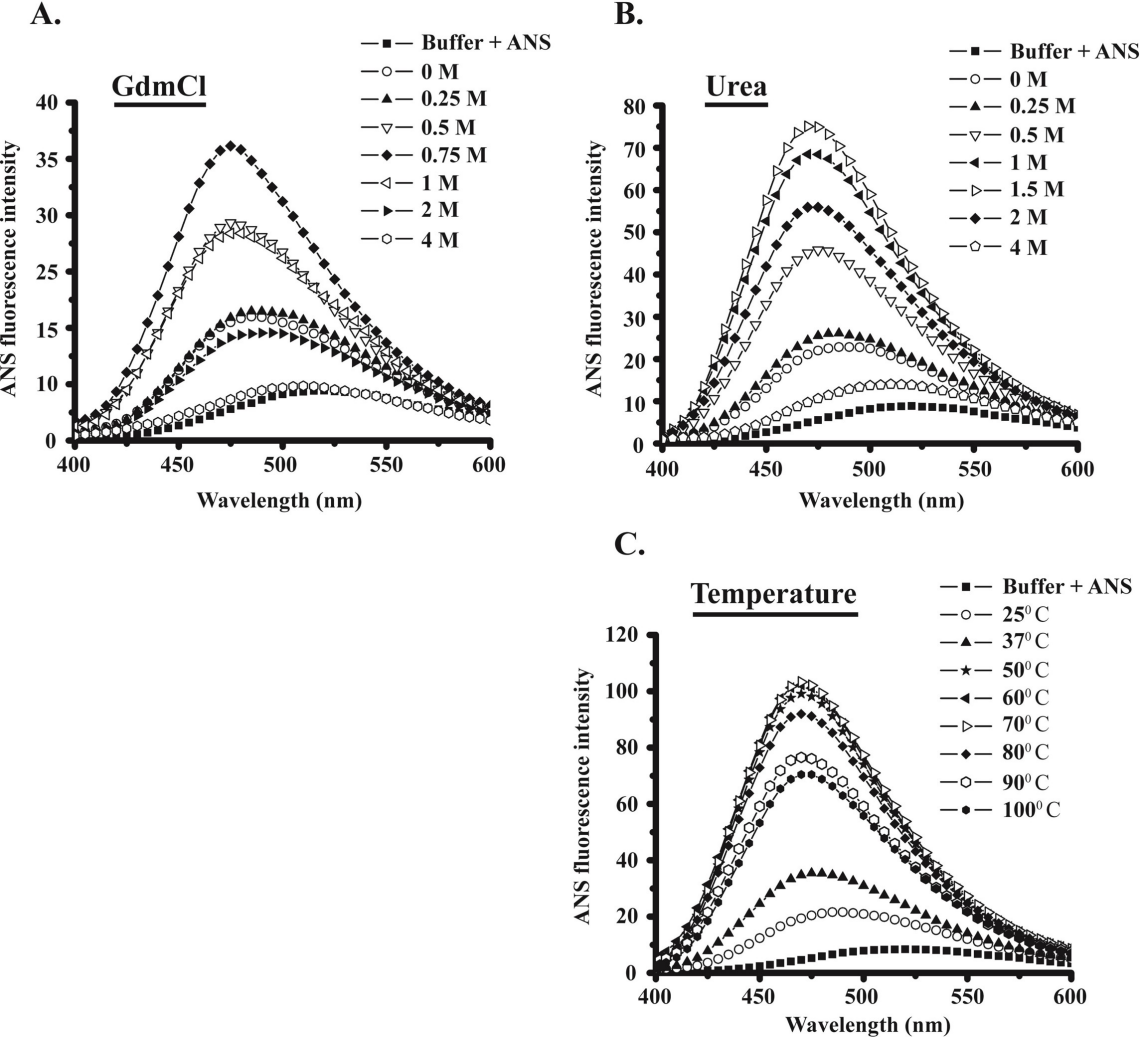
Exposure of hydrophobic patches at intermediate state of unfolded MsEis analyzed by ANS fluorescence. Changes in ANS emission spectra (ʎ_max_ = 518 nm) of MsEis protein in 20 mM phosphate buffer (pH 8.0) as a function of GdmCl **(Panel A)**, urea **(Panel B)** and temperature **(Panel C)**. Acquisition of ANS emission spectra was performed at 25°C, strictly in dark after incubating protein sample and ANS dye in 1:5 molar ratios for 5 min.

During GdmCl induced unfolding of MsEis, maximum ANS fluorescence intensity was observed at 0.75 M GdmCl corresponding to the formation of intermediate state as detected in CD analysis of GdmCl-treated MsEis (Figs 4A and 1C). Similarly, urea induced unfolding of MsEis also showed highest ANS fluorescence intensity in the presence of 1.5 M urea, at which intermediate state formation was observed in CD analysis of urea-treated MsEis protein (Figs 4B and 1D). Since 4M of either GdmCl or urea induces complete unfolding of MsEis protein, the binding of ANS to MsEis was also diminished at the respective concentrations of these denaturants (Figs 4A and 1B).

ANS binding assay with MsEis was also performed at different temperatures ranging from 25°C to 100°C. The maximum ANS fluorescence intensity obtained at 70°C indicated the presence of partially unfolded intermediates which was also observed using CD at same temperature during thermal unfolding studies (Figs 4C and 1B). However, the ANS fluorescence intensity was not completely decreased even at 100°C. This result indicates that temperature only induces the formation of a non-native form of MsEis protein and does not unfold it completely.

### Oligomeric forms in MsEis are not SDS-resistant

**Fig 5:**
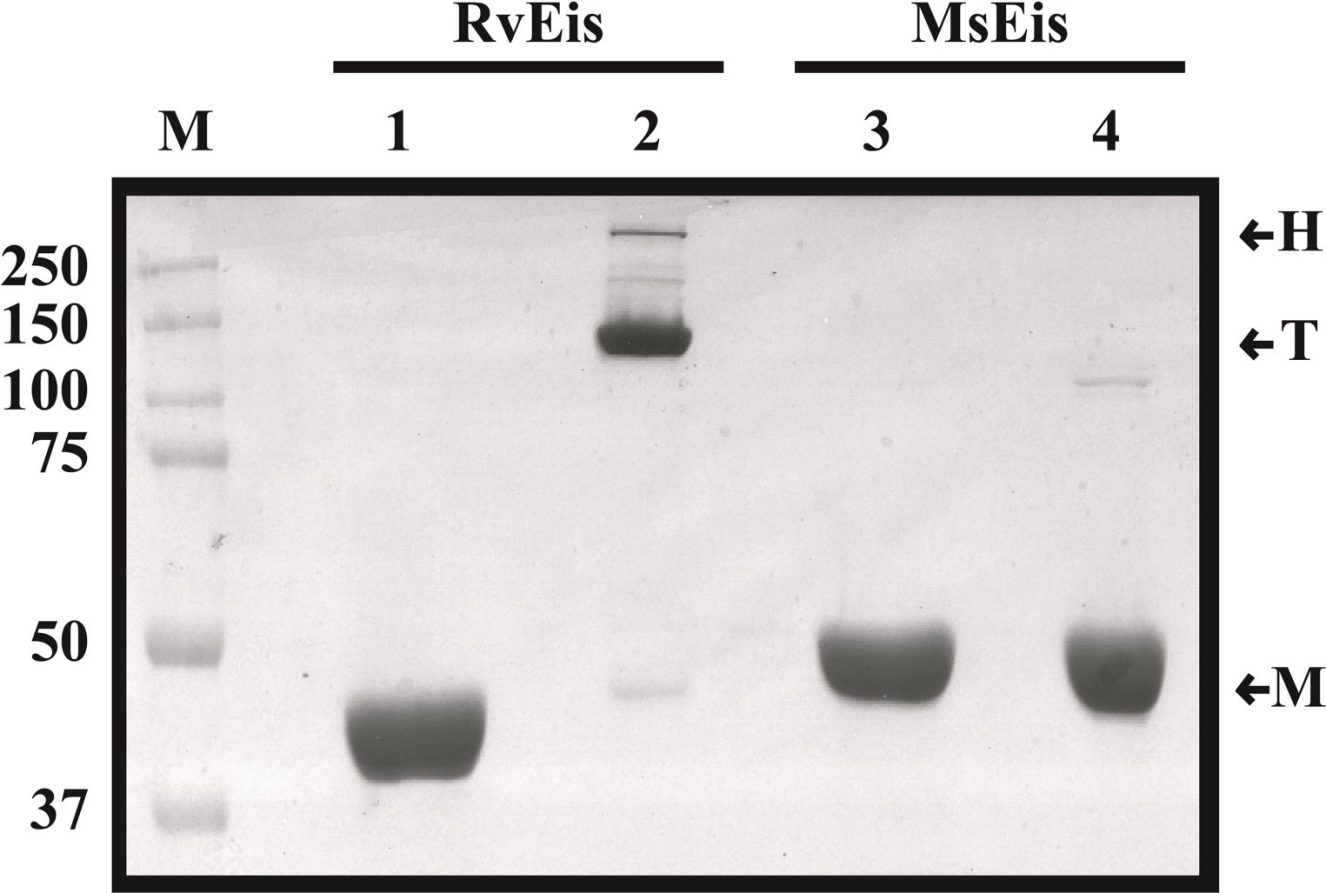
SDS-PAGE assay of RvEis and MsEis proteins. Equal amount of boiled and unboiled RvEis and MsEis proteins were electrophoresed on 8% SDS-PAGE gel. Lane 1- boiled RvEis, lane 2- unboiled RvEis, lane 3- boiled MsEis and lane 4- unboiled MsEis. H- Hexamer, T- Trimers and M- Monomers. Markers are shown on the left side of the figure.

RvEis is known to be a SDS-resistant protein [13]. In order to determine if MsEis also exhibits a similar property, equal amounts of boiled and unboiled RvEis or MsEis proteins were analyzed using SDS-PAGE assay. As expected, three forms corresponding to the hexamer (H), trimer (T) and monomer (M), were observed in unboiled fraction of RvEis but unboiled MsEis protein mainly existed only in monomeric form (Fig 5A, lanes 2 vs 4). Both the proteins exist only in the monomeric forms after boiling (Fig 5A, lane 1 and lane 3). The calculated molecular weights of N–terminal His tagged RvEis and MsEis are 47.96 kDa and 47.72 kDa, respectively. Surprisingly, MsEis exhibits a slower electrophoretic mobility in comparison to RvEis. We could not decipher the reason behind this property.

### Comparison of SDS-induced structural changes in MsEis and RvEis proteins

**Fig 6:**
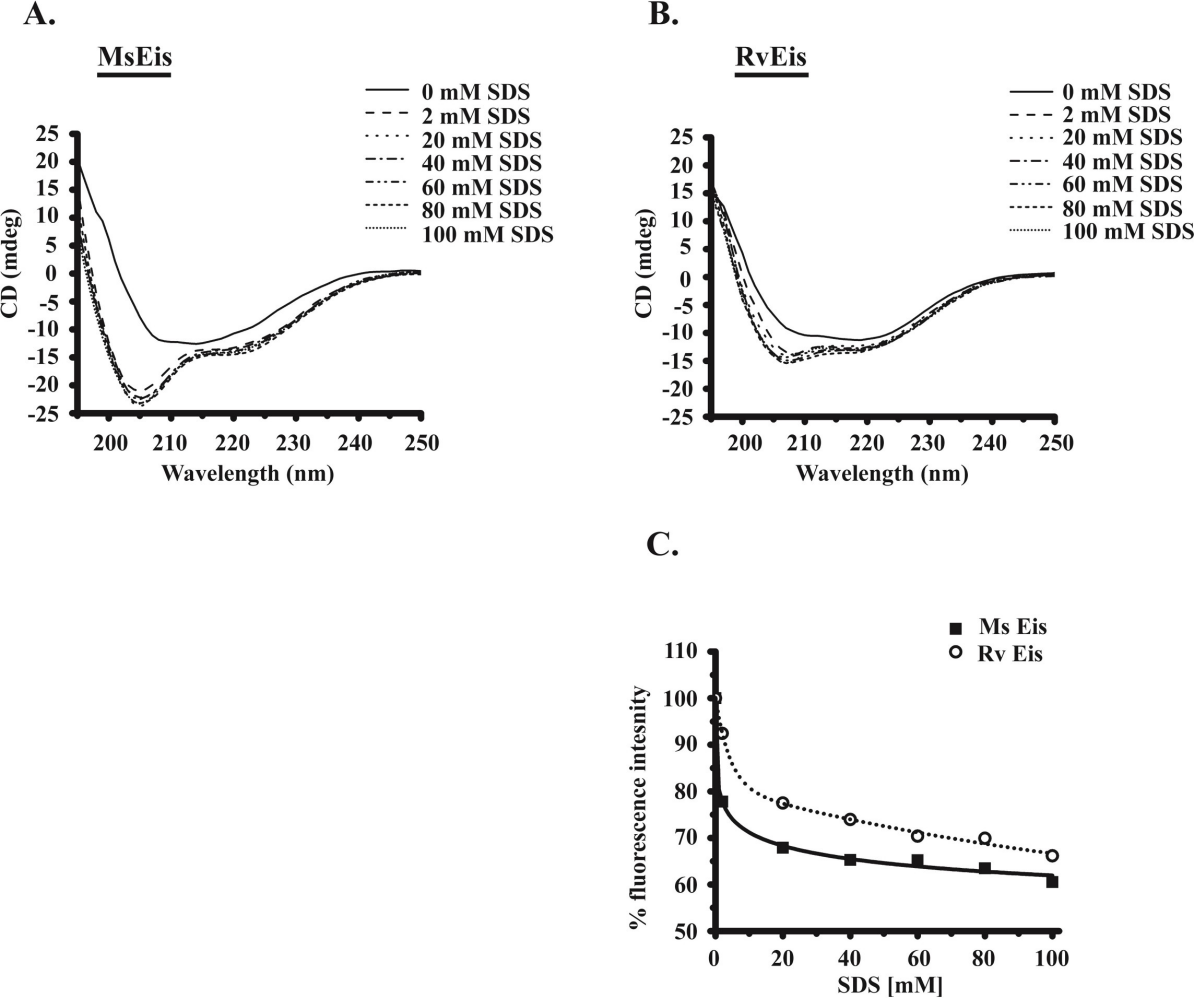
Comparison of SDS resistance ability of MsEis and RvEis proteins. Changes in the secondary structure of MsEis **(Panel A)** and RvEis **(Panel B)** proteins were monitored by following changes in the far-UV CD spectra recorded from 250 nm to 195 nm at increasing concentration of SDS. SDS induced changes in conformation of MsEis and RvEis proteins were monitored by analyzing changes in the intrinsic tryptophan fluorescence intensity at emission maximum (337 nm) and the values of untreated Eis protein were taken as 100% **(Panel C)**.

The oligomeric forms in MsEis protein were not found to be SDS resistant; therefore, we checked the SDS induced structural changes in MsEis and RvEis proteins. Far-UV CD spectra of MsEis in the presence of increasing concentration of SDS exhibited significant increase in the CD ellipticity at 2 mM SDS with a shift in negative peak from 208 nm to 205 nm (Fig 6A). In the presence of 20 mM SDS, CD ellipticity of MsEis was further increased; however no changes were observed beyond 20 mM SDS (Fig 6A). A similar increase in CD ellipticity was also observed in case of RvEis after treating with 2 mM SDS with a concomitant shift in negative peak to 205 nm. However, no substantial changes were seen in case of RvEis beyond 2 mM SDS (Fig 6B). Moreover, an increase in CD ellipticity from -11 mdeg at 208 nm to -24 mdeg at 205 nm was observed in MsEis (Fig 6A) whereas in RvEis an increase from -10 mdeg at 208 nm to -15 mdeg at 205 nm was recorded (Fig 6B).

The changes in intrinsic tryptophan fluorescence spectra of MsEis and RvEis proteins were also determined in the presence of increasing concentration of SDS. At 2 mM SDS, ~ 95% and ~ 80% fluorescence intensities were retained in RvEis and MsEis proteins, respectively, which further decreased to ~ 70% and ~ 65% at 100 mM SDS (Fig 6C).

### Comparison of thermostability of MsEis and RvEis proteins

**Fig 7:**
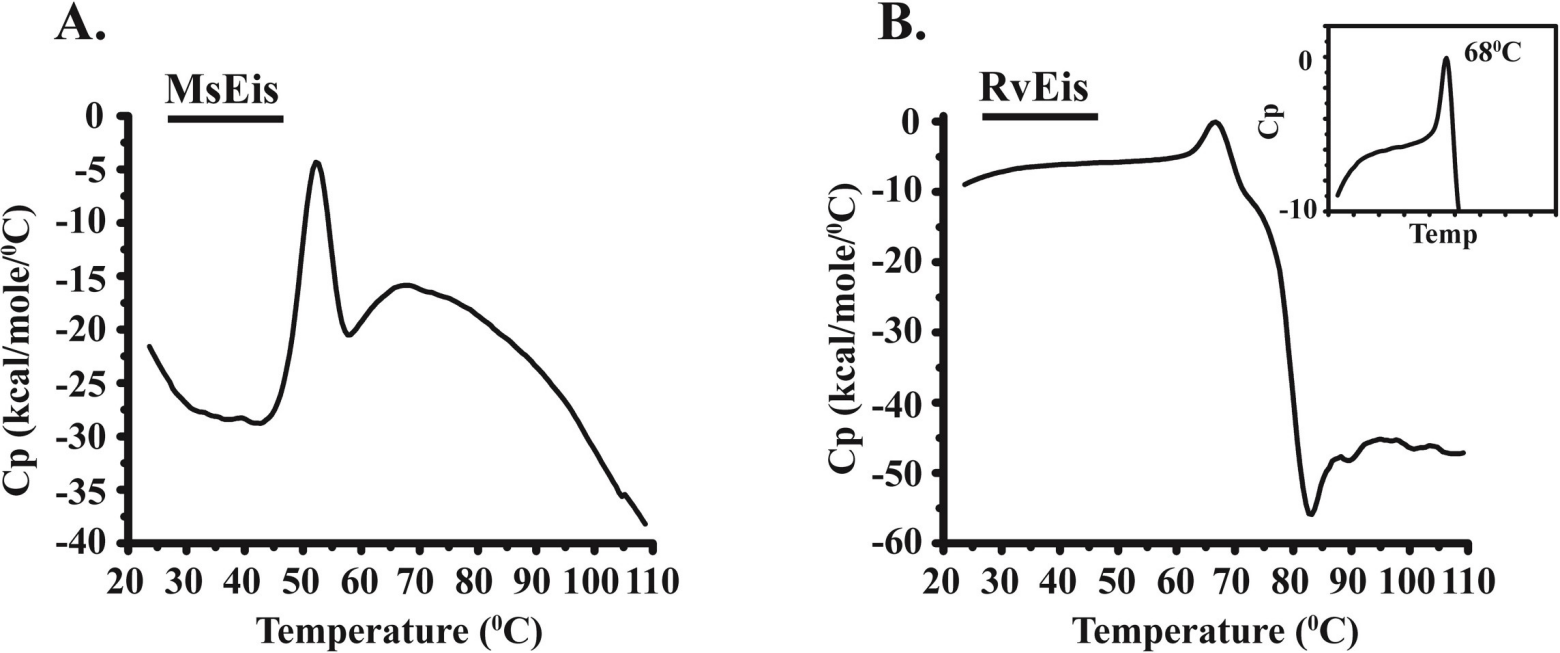
Comparison of temperature stability of MsEis and RvEis proteins analyzed by DSC. Raw DSC thermograms show changes in the molar heat capacity of MsEis **(Panel A)** and RvEis **(Panel B)** proteins at increasing temperature from 20°C to 110°C. The protein concentration was 0.25 mg/ml for each experiment. The transition curve in both the cases is shown after the subtraction of buffer reference and normalization with the concentration used for measurement. Inset shows the zoomed view of endothermic peak of RvEis.

Differential Scanning Calorimetry (DSC) was used to compare the thermal stability of MsEis and RvEis proteins in 20 mM sodium phosphate buffer (pH 8.0). The DSC thermogram of MsEis displayed a sharp peak, with the maximum heat capacity (T_max_) at temperature 52°C, and a broad peak (T_max_ ~ 70°C) in the temperature range of 60°C to 95°C (Fig 7A). The first peak is assigned to partial unfolding of MsEis and the second broad peak indicates the formation of soluble aggregates which corroborates the results of ANS binding to MsEis protein at temperature ranging from 60°C to 80°C. On the contrary, RvEis protein also exhibited a single endothermic peak having maximum heat capacity change at temperature (T_max_) ~ 70°C associated with protein unfolding followed by exothermic aggregation at ~ 80°C (Fig 7B). It is interesting to note that the thermal denaturation of both MsEis and RvEis proteins at the same scan rates are calorimetrically irreversible and heating RvEis protein to 110°C results in visible precipitation whereas MsEis protein forms soluble aggregates without any precipitation at 110°C.

### Scan-rate dependence of MsEis and RvEis thermal denaturation

**Fig 8:**
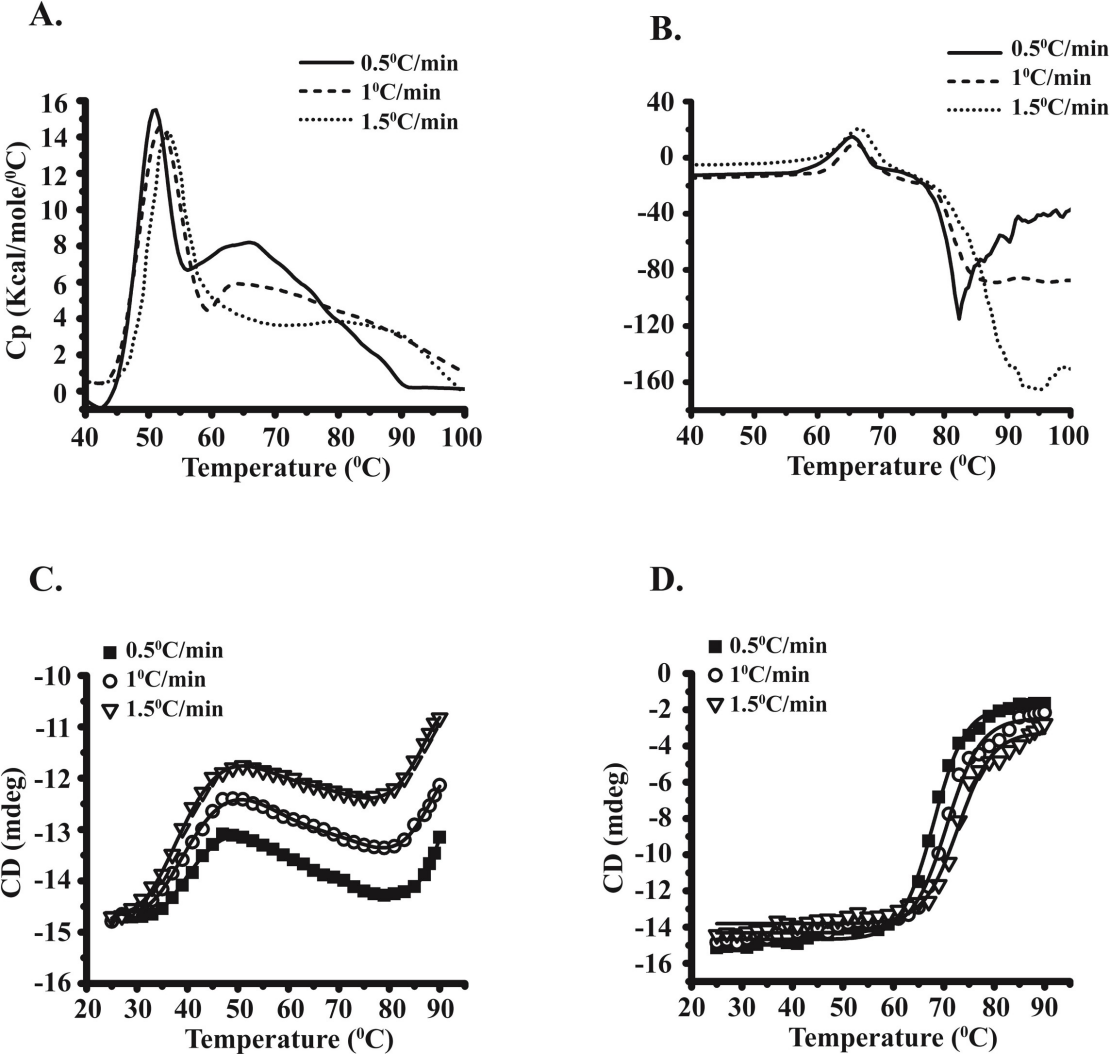
Temperature dependence of heat flow of MsEis and RvEis proteins analyzed by DSC and CD spectroscopy. Raw DSC thermograms of MsEis **(Panel A)** and RvEis **(Panel B)** after subtraction of buffer baseline and normalization with protein concentration at different scanning rates, i.e., 0.5°C/min, 1°C/min and 1.5°C/min. Thermal melting curve of MsEis **(Panel C)** and RvEis **(Panel D)** at different scanning rates as monitored by the changes in ellipticity at 222 nm using CD spectroscopy.

The thermal denaturation of both MsEis and RvEis was found to be calorimetrically irreversible; therefore, it would be interesting to know if thermal denaturation is under kinetic control. To test it, the DSC and CD experiments were performed using same protein concentration of MsEis and RvEis proteins at different scanning rates i.e. 0.5°C/min, 1°C/min and 1.5°C/min. With increase in scanning rate, changes in the shape of DSC thermograms of MsEis and RvEis were observed and the T_max_ was shifted to higher temperature (Table 1), thus indicating that the irreversible thermal denaturation was kinetically controlled (Fig 8A and 8B). During the slow heating (0.5°C/min) of MsEis, relatively larger changes in the heat capacity at ~ 65°C were seen indicating a higher abundance of aggregates (Fig 8A). In case of RvEis, the exothermic effect (the rate of aggregation) was also dependent on the scanning rate (Fig 8B).

**Table 1:** T_max_ and T_m_ values of MsEis and RvEis at different scanning rate as analyzed by DSC and CD.

	DSC		CD	
Scanning rate (°C/min)	T_max_ (°C)	T_max_ (°C)	T_m_ (°C)	T_m_ (°C)
	MsEis	RvEis	MsEis	RvEis
0.5°C/min	51.08	65.27	47	68
1°C/min	52.17	66.01	49	72
1.5°C/min	53.11	66.88	51	73

Similarly, the effect of different scanning rate on thermal denaturation was also studied by monitoring changes in the ellipticity at 222 nm using CD spectroscopy. While MsEis exhibited three-state unfolding process with the formation of a stable intermediate state, RvEis exhibited two-state denaturation curve. The MsEis protein demonstrated less change in the ellipticity at 90°C, whereas a complete loss of ellipticity was seen in case of RvEis. The thermal transition curves of MsEis and RvEis varied with changes in the scanning rates (Fig 8C and 8D). With increase in scanning rate, Tm was increased for both the Eis proteins (Table 1).

### Oligomeric status of unfolding intermediates in MsEis protein

**Fig 9:**
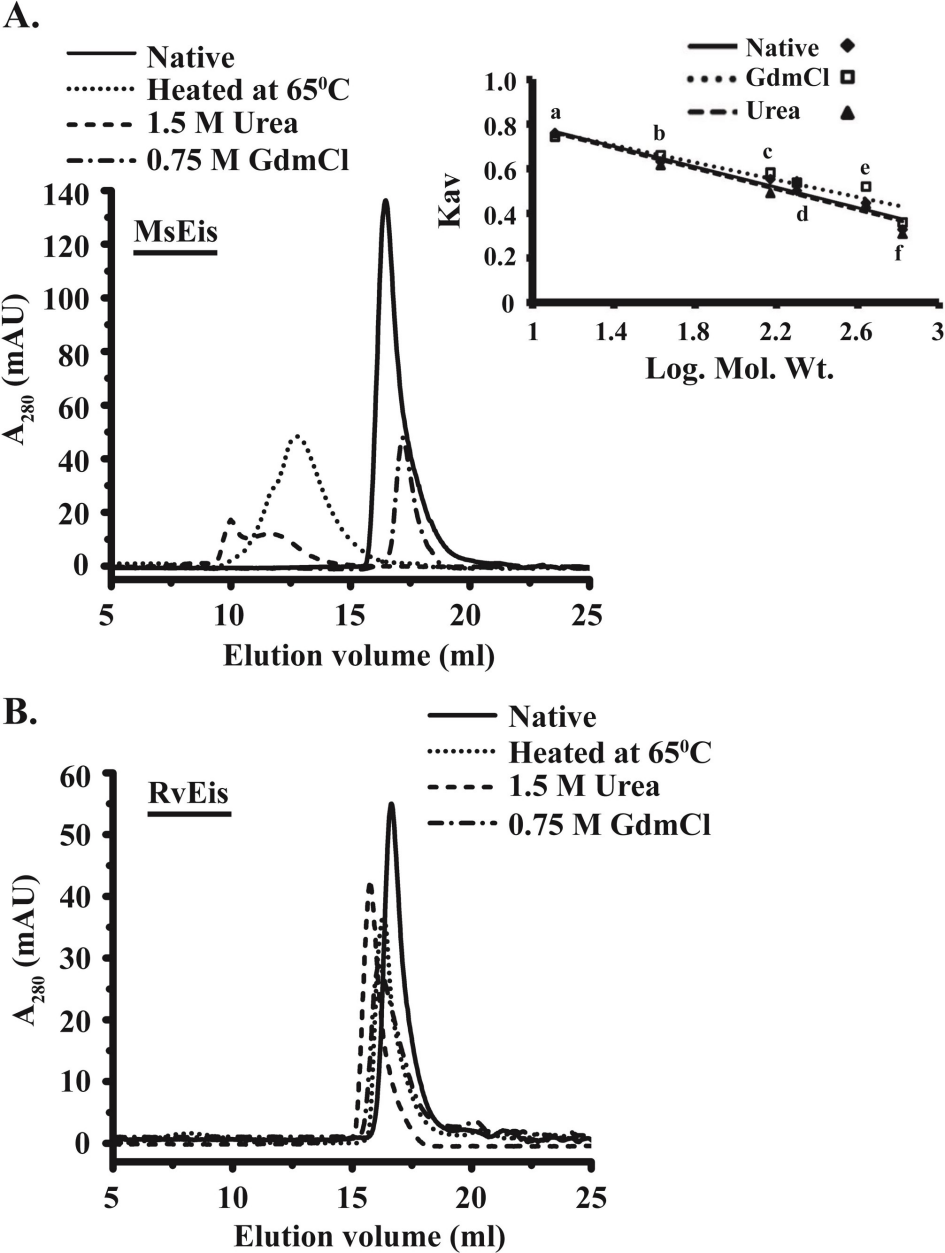
Oligomeric status of MsEis and RvEis proteins during unfolding induced by GdmCl, urea and temperature. Size Exclusion Chromatographic (SEC) elution profiles of MsEis **(Panel A)** and RvEis **(Panel B)** proteins treated with 0.75 M GdmCl (short dash dot), 1.5 M urea (short dash) and heated at 65°C (short dot) in comparison to untreated protein sample (solid) loaded on Superose 6 Increase 10/300 GL column pre-equilibrated with 10 mM Tris-Cl pH 8.0, 150 mM NaCl, 1 mM EDTA and 0.5 mM β-ME. The standard curve prepared by using different molecular weight markers run either in the native conditions (filled diamond), or 0.75M GdmCl (open square) or 1.5 M urea (filled triangle). a: Cytochrome c (12.9 kDa); b: Ovalbumin (43 kDa); c: Alcohol dehydrogenase (150 kDa); d: β-amylase (200 kDa); e: Ferritin (440 kDa) and f: thyroglobulin (669 kDa).

Size exclusion chromatography was used to analyze the oligomeric status of intermediate population appeared during temperature, urea and GdmCl induced unfolding of MsEis protein. A broad peak of higher molecular weight (>600 kDa) with elution volume of 12.81 ml was observed when the MsEis was incubated at 65°C for 10 min, whereas untreated MsEis eluted as hexamer (~ 295 kDa) (Fig 9A). Moreover, treatment of MsEis with 1.5 M urea led to the formation of aggregates of two types: aggregates with high molecular weight which eluted in void volume and a small amount of low molecular weight aggregates (>700 kDa) which eluted at 11.65 ml elution volume. In case of 0.75 M GdmCl treatment, MsEis exhibited a sharp peak corresponding to ~ 280kDa with elution volume of 17.19 ml, suggesting the presence of only hexamers (Fig 9A).

When RvEis protein was treated with denaturants under similar conditions, formation of aggregates was not observed. The elution volume of 16.66 ml for native hexameric RvEis decreased to 16.31 ml, 16.10 ml and 15.76 ml upon heating at 65°C, treatment with 0.75 M GdmCl and 1.5 M urea, respectively (Fig 9B). The minor changes in elution volume indicate a change in hydrodynamic radius upon treatment with various denaturants.

### Comparison of acetyltransferase activity of MsEis and RvEis

**Fig 10:**
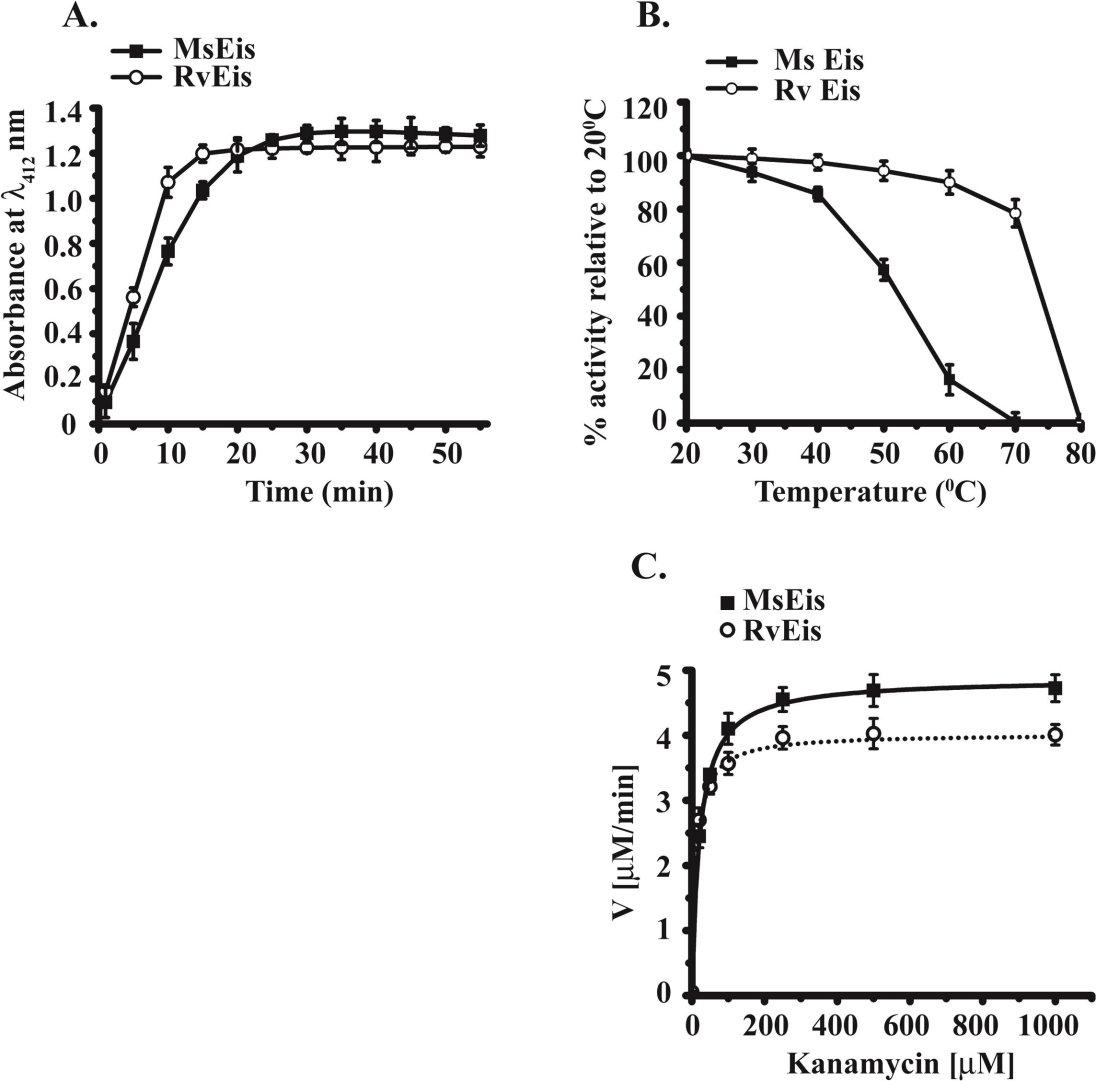
Time kinetics, temperature stability and steady-state kinetic parameters of KAN acetylation by MsEis and RvEis. Time course of changes in the absorbance at 412 nm of NTB^-^ ions formed as a result of acetyltransferase activity of MsEis and RvEis proteins **(Panel A)**. The effect of temperature on the catalytic activity of MsEis and RvEis was determined after incubating the Eis proteins at different temperatures ranging from 20°C to 80°C **(Panel B)**. Kinetic parameters were calculated for the acetylation of KAN by MsEis and RvEis at fixed concentration of acetyl CoA and increasing concentration of KAN antibiotic **(Panel C)**. The experimental values were fitted to Michaelis-Menten equation using OriginPro 8.0 software (Origin Labs, Northampton, USA).

Time course changes in acetyltransferase activity of MsEis and RvEis proteins were determined using kanamycin as substrate. MsEis protein was found to have 1.3 fold decreased rate of product formation in comparison to RvEis. Moreover, RvEis protein exhibited a rapid increase in the absorbance of TNB^-^ ions with time, a product formed as a result of acetylation of kanamycin and attained a plateau at 15 min. MsEis exhibited a slower rate of increase in absorbance of TNB^-^ and plateau was attained after 25 min (Fig 10A).

The effect of temperature on acetyltransferase activity of both the proteins was also evaluated. The MsEis protein was found to be only ~ 60% active at 50°C with complete loss of activity at 70°C whereas RvEis was almost 80% active even at 70°C and was completely inactive at 80°C (Fig 10B).

Furthermore, Michaelis-Menten kinetic parameters were determined to further explicate the differences in the action of MsEis and RvEis proteins. A hyperbolic curve was obtained when a fixed concentration of acetyl CoA and varying concentrations of kanamycin were used (Fig 10C). The calculated K_m_ value of 21 μM and 11 μM for kanamycin in case of MsEis and RvEis proteins, respectively, revealed ~ 2.0 fold higher aminoglycoside binding affinity in case of RvEis protein than MsEis. Moreover, ~ 1.5 fold decrease in the catalytic efficiency was observed for MsEis when compared to RvEis (Table 2).

**Table 2:** Comparison of steady-state kinetic parameters for KAN acetylation by MsEis and RvEis proteins.

	K_m_ (μM)	k_cat_(s^-1^)	V_max_ (μM.min^-1^)	k_cat_/K_m_ (M^-1^.s^-1^)
**MsEis**	**21 ± 0.1**	**0.027 ± 0.005**	**4.8 ± 0.11**	**1274 ± 22**
**RvEis**	**11 ± 1.6**	**0.022 ± 0.002**	**4.0 ± 0.20**	**2143 ± 27**

All kinetic parameters are given in mean ± S.D.

### Unlike RvEis, overexpression of MsEis does not provide thermal protection to *M*. *smegmatis*

**Fig 11:**
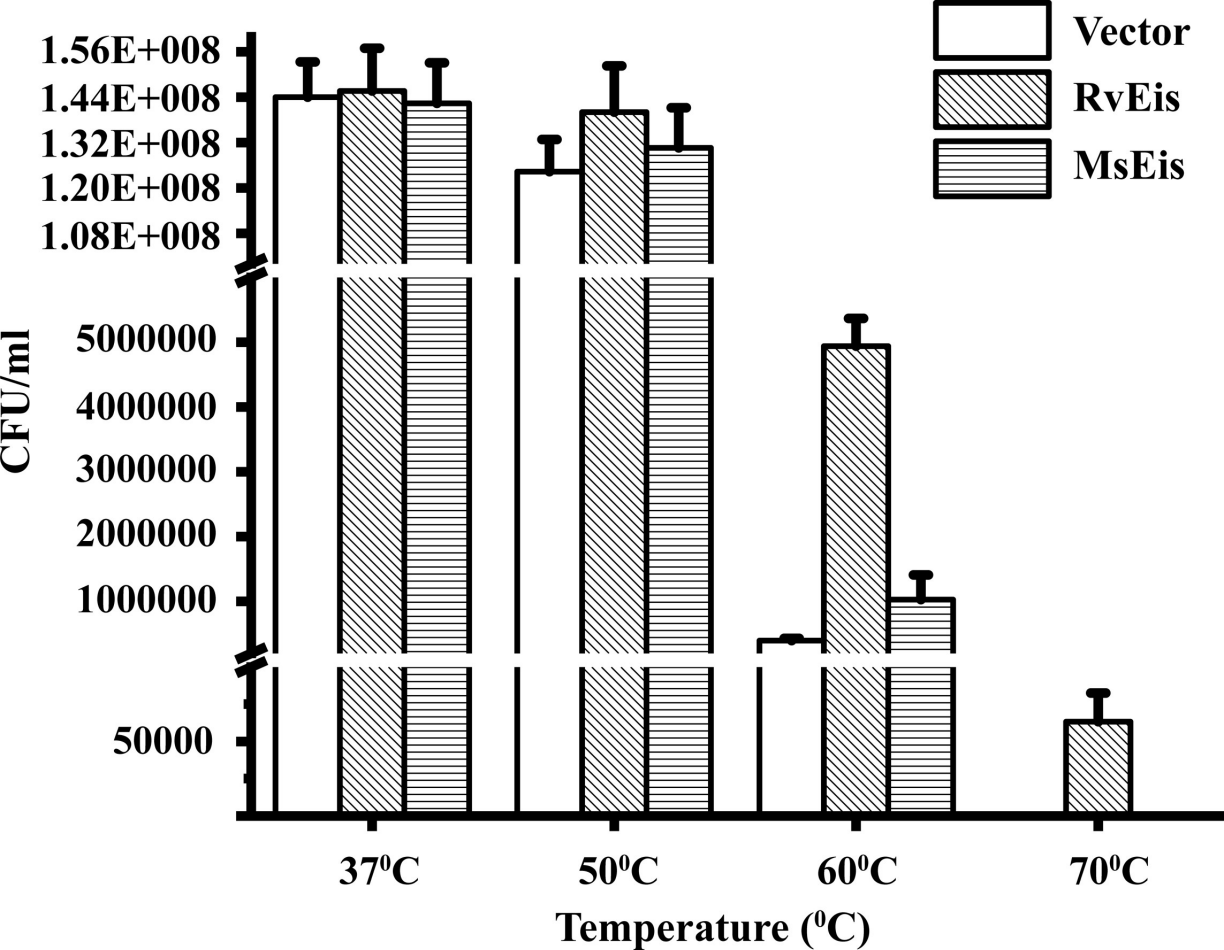
Survival profile of *M*. *smegmatis* harboring *eis* gene from *M*. *tuberculosis* and *M*. *smegmatis* at different temperatures. *M*. *smegmatis* bacterial cells grown to log phase at 37°C were incubated at 37°C, 50°C, 60°C and 70°C for 10 min followed by plating of appropriate dilutions on 7H11 agar. Absolute CFU counts at various temperatures obtained from three independent experiments were plotted as mean ± standard deviation.

The thermal protection rendered by RvEis and MsEis proteins was compared by heating *M*. *smegmatis* bacterial cultures overexpressing either RvEis or MsEis at different temperatures followed by CFU assay. *M*. *smegmatis* culture transformed with vector alone was included as a negative control. At 37°C, the CFU count was similar for all the samples. However, a marginal loss in CFU for bacteria harboring vector alone and MsEis protein in comparison to RvEis was observed at 50°C (Fig 11). At 60°C, in comparison to *M*. *smegmatis* cells harboring vector alone, MsEis overexpressing bacterial cells demonstrated only ~1.6 fold increased CFU counts, whereas, the bacterial cells overexpressing RvEis exhibited ~12 fold increase in the survival. Upon heating at 70°C, the viability of mycobacterial cells for all the cultures were less, nevertheless, the CFU counts obtained upon RvEis overexpression were better than the negligible CFU counts observed for bacteria overexpressing MsEis and vector alone (Fig 11).

## Discussion

Eis is an aminoglycoside acetyltransferase enzyme which catalyzes the acetylation of aminoglycoside drugs and drives them inactive [7,8]. Both RvEis and MsEis proteins exist as hexamers composed of two symmetric trimers and possess similar structure and function [9,12]. Acetyltransferase activity is a characteristic of hexameric form of RvEis while the monomers and trimers are inactive [13]. RvEis hexamers are SDS-resistant with a high structural stability and follow a two-state process of unfolding [13]. Although structural characterization of RvEis has been reported, such structural details about MsEis remain elusive. Therefore, we embarked upon the characterization of structure stability, acetyltransferase activity and unfolding behavior of MsEis.

Unlike RvEis, MsEis was found to be highly susceptible to chemical denaturants and unfolds completely at low concentration of GdmCl and urea. The increased red shift in tryptophan fluorescence emission maxima of MsEis at early concentration of chemical and thermal denaturants indicates that the tryptophan residues in MsEis are exposed to a polar microenvironment and MsEis has reduced conformational stability than RvEis. The correlation of red shift in emission spectrum with structural unfolding or conformational changes in proteins is well known [23,24]. The thermostability of MsEis protein was also found to be less than RvEis. In both Eis proteins, the thermal denaturation is kinetically controlled and thermodynamically irreversible as no thermal effect was observed during reheating scans. The scan-rate dependence of irreversible thermal denaturation have been reported earlier during unfolding of Thermolysin enzyme from *Bacillus thermoproteolyticus rokko* [25].

As a function of increasing temperature, MsEis undergoes a partial conformational unfolding with a concomitant formation of soluble aggregates whereas in case of RvEis, a complete thermal unfolding was observed followed by an exothermal effect resulting in the formation of visible precipitates. A similar pattern of exothermal aggregation preceded by conformational unfolding has also been shown for *E*. *coli* Maltodextrin Glucosidase [26].

In contrast to RvEis, the oligomers in MsEis were found to be SDS sensitive. Although, SDS treatment triggered minimal changes in the tertiary structure of both MsEis and RvEis proteins as deduced from fluorescence emission spectra, the increase in α-helical content in MsEis protein induced by SDS treatment was higher than that observed for RvEis as marked by enhanced CD ellipticity in far-UV CD spectra. Similar changes in α-helical content with increasing concentration of SDS have also been reported for Soyabean Glycinin [27], Papain [28] and Bovine Serum Fetuin [29]. Unlike RvEis, MsEis unfolding follows a biphasic denaturation pattern indicating the formation of a partially unfolded intermediate state. The intermediate state of MsEis has native protein like secondary structure (as evidenced by Far-UV CD Spectra), reduced conformational stability (shown by intrinsic tryptophan fluorescence studies) and an exposed hydrophobic surface area (analyzed by ANS binding assay). These are the characteristic features of a molten-globule intermediate [30,31]. Thus, the intermediate phase observed during unfolding of MsEis could be attributed to the formation of molten-globule like structure. A strong affinity of ANS for molten globule like unfolding intermediates relative to their native or denatured state has also been seen in Peanut Lectin [32], *Bacillus amyloliquefaciens* α-amylase [33] and sheep serum albumin [34].

Further, the molten-globule like intermediate state of MsEis was found to have a higher molecular weight than native protein as determined using size exclusion chromatography. These results suggest that unfolding of MsEis is accompanied by irreversible self-association of partial folded oligomers resulting in the formation of soluble aggregates. Inactivated G-actin [35] and Porcine growth hormone [36] are known to have self-association of equilibrium unfolding intermediates upon treatment with chemical denaturants and low pH, respectively. The aggregates formation was not observed when MsEis treated with GdmCl and only hexameric forms were seen. It can be due to very less stability of soluble aggregates of MsEis formed upon GdmCl treatment.

There are contrasting reports on the comparison of aminoglycoside acetyltransferase activity of RvEis and MsEis. While RvEis is reported to have higher catalytic efficiency for KAN acetylation than MsEis as reported by Garneau-Tsodikova group [6,10,11], a report by Kim and group states that MsEis has a partially increased binding affinity towards KAN with higher catalytic efficiency than the RvEis [12]. Due to this disparity, we compared the catalytic efficiency of RvEis and MsEis proteins. Interestingly, our results are in contrast to those reported by Kim and group [12] and support the findings of Garneau-Tsodikova’s group. We observed a slower rate of kanamycin acetylation, the weaker binding affinity for kanamycin and a lesser catalytic efficiency in case of MsEis protein than the RvEis.

RvEis protein is known to enhance the survival of *M*. *smegmatis* at high temperatures [13]. Enhanced survival of *M*. *smegmatis* overexpressing RvEis in comparison to wild type bacteria expressing only endogenous levels of MsEis prompts an investigation into the relative effect of MsEis versus RvEis overexpression on the survival of *M*. *smegmatis*. However, the overexpression of MsEis failed to yield thermal protection to *M*. *smegmatis* when compared to bacteria overexpressing RvEis protein thus demonstrating the incapability of MsEis in providing thermal resistance to *M*. *smegmatis*. In crux, the present work sets an example of a comparative study of protein homologs from pathogenic and non-pathogenic bacteria that show different unfolding behavior, structural stability and catalytic activities despite having high sequence identity and similar oligomeric organization. However, the reason for the differential behavior of MsEis and RvEis protein needs to be further investigated.

## Supporting information

S1 Fig Expression and purification of MsEis protein. Purification of His tagged MsEis using Ni-NTA affinity chromatography. Lanes 1–4 show uninduced, induced, supernatant and flow through fractions respectively. Lanes 5–9 show fractions after washing with 10, 20, 30, 40 and 50 mM imidazole containing phosphate buffer **(Panel A)**. Lanes 1–9 show eluted fractions in 250 mM imidazole in phosphate buffer **(Panel B)**. Lane Mr in both panels shows standard molecular weight markers.
(TIF)

S2 Fig Comparison of amino acid sequences of MsEis and RvEis. The amino acid sequences of MsEis and RvEis proteins were compared using Clustal Omega software (https://www.ebi.ac.uk/Tools/msa/clustalo/). All the tryptophan residues are highlighted with rectangles. MsEis has six tryptophan residues (W15, W38, W178, W193, W251 and W293) and RvEis possess seven (W13, W36, W182, W197, W253, W289, W295). Out of seven tryptophan residues present in RvEis, six are conserved in MsEis protein. The tryptophan W289 which is not conserved in MsEis is indicated by an arrow.
(TIF)

S1 Table Raw CD ellipticity data corresponding to Fig 1A–1D. CD ellipticity data has been shown in MRE for Panel A and in mdeg units for Panel B. The percent of protein folded for Panels C and D was calculated as described in material and methods and shown with respect to increasing concentration of either GdmCl or Urea.
(XLSX)

S2 Table Raw data corresponding to Fig 2A–2G. Percentage values of fluorescence intensity are shown for the unfolding and refolding of MsEis protein induced by either urea or GdmCl at different time intervals.
(XLSX)

S3 Table Raw data corresponding to Fig 3A–3F. The percent fluorescence intensity for Panels A, C and E and emission maxima values for Panels B, D and F are shown for the denaturation of MsEis and RvEis proteins induced by GdmCl, urea and temperature.
(XLSX)

S4 Table Raw data of ANS fluorescence intensity corresponding to Fig 4A–4C. ANS fluorescence intensity values (in arbitrary units) of MsEis protein treated with increasing concentration of GdmCl, urea and heating at various temperatures.
(XLSX)

S5 Table Raw data corresponding to Fig 6A–6C. CD spectra values in mdeg for Panels A and B and percent fluorescence intensity for Panel C of MsEis and RvEis proteins in the presence of varying concentration of SDS.
(XLSX)

S6 Table Raw data collected in DSC experiment corresponding to Fig 7A and 7B. DSC values of MsEis and RvEis proteins shown in kcal/mol/°C.
(XLSX)

S7 Table Raw data corresponding to Fig 8A–8D. DSC data for Panels A and B and CD ellipticity data for Panels C and D at different scanning rates.
(XLSX)

S8 Table Raw data corresponding to Fig 9A and 9B. Size exclusion chromatography data of MsEis and RvEis proteins in the presence of GdmCl or urea or native conditions.
(XLSX)

S9 Table Raw data corresponding to Fig 10A–10C. The absorbance values (at λ_412_ nm) of NTB- ions released as a result of acetylation of kanamycin substarte by MsEis and RvEis proteins.
(XLSX)

S10 Table Raw data corresponding to Fig 11. CFU values of *M*. *smegmatis* overexpressing either vector or MsEis or RvEis and heated at various temperatures.
(XLSX)
