# Supplementary figures and images for: Differential thermal stability, conformational stability and unfolding behavior of Eis proteins from Mycobacterium smegmatis and Mycobacterium tuberculosis
Source: PLoS One. 2019 Mar 25;14(3):e0213933. doi: 10.1371/journal.pone.0213933 (PMC6433294; doi:10.1371/journal.pone.0213933)

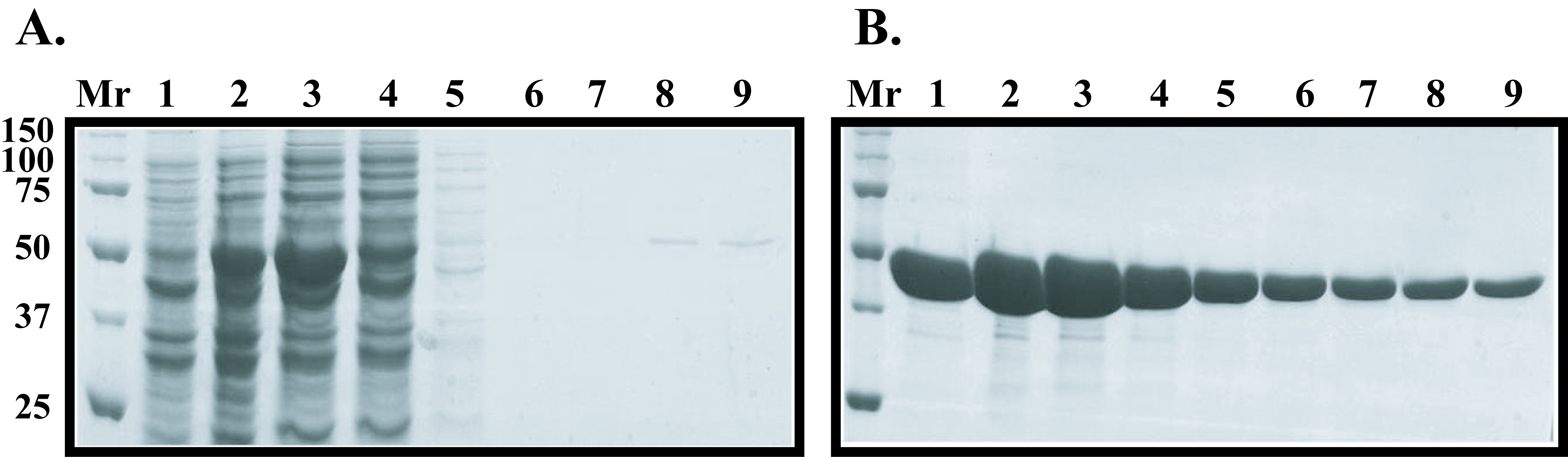

Supplement: S1 Fig — Purification of His tagged MsEis using Ni-NTA affinity chromatography. Lanes 1–4 show uninduced, induced, supernatant and flow through fractions respectively. Lanes 5–9 show fractions after washing with 10, 20, 30, 40 and 50 mM imidazole containing phosphate buffer (Panel A). Lanes 1–9 show eluted fractions in 250 mM imidazole in phosphate buffer (Panel B). Lane Mr in both panels shows standard molecular weight markers. (TIF) [file pone.0213933.s001.tif]

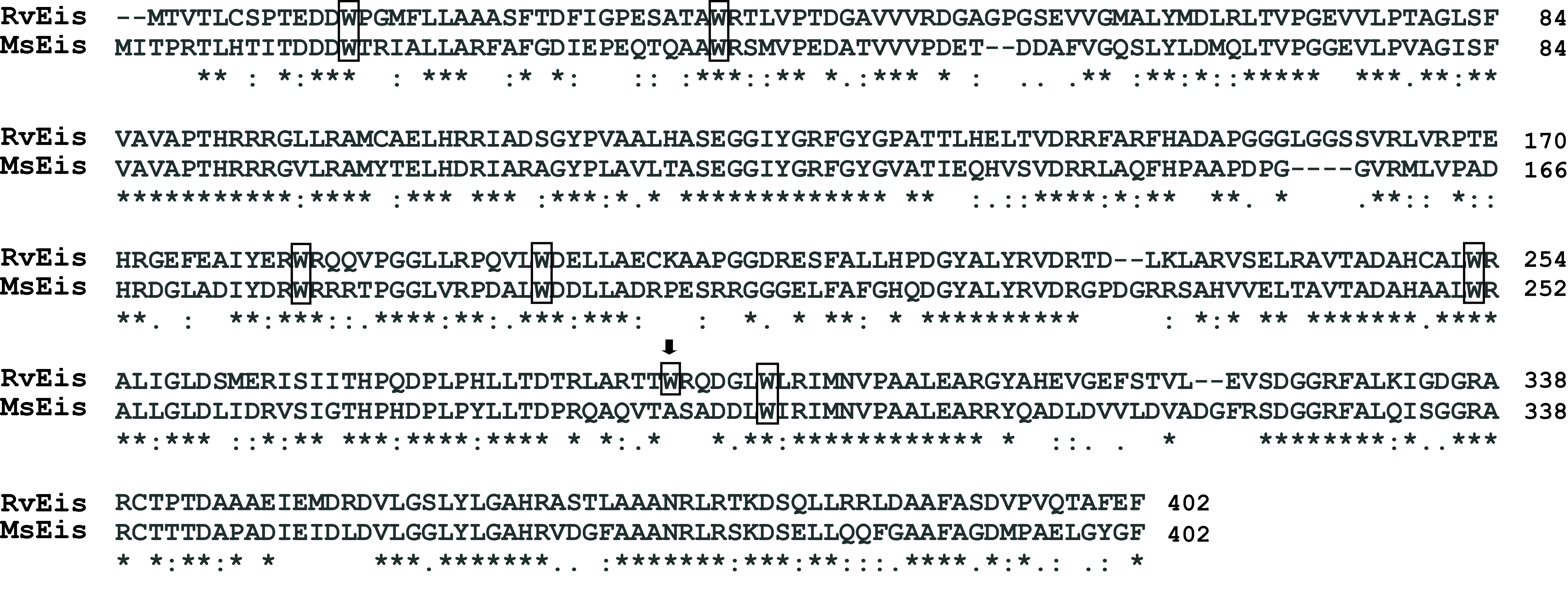

Supplement: S2 Fig — The amino acid sequences of MsEis and RvEis proteins were compared using Clustal Omega software (https://www.ebi.ac.uk/Tools/msa/clustalo/). All the tryptophan residues are highlighted with rectangles. MsEis has six tryptophan residues (W15, W38, W178, W193, W251 and W293) and RvEis possess seven (W13, W36, W182, W197, W253, W289, W295). Out of seven tryptophan residues present in RvEis, six are conserved in MsEis protein. The tryptophan W289 which is not conserved in MsEis is indicated by an arrow. (TIF) [file pone.0213933.s002.tif]
